# Hypothesis Relating the Structure, Biochemistry and Function of Active Zone Material Macromolecules at a Neuromuscular Junction

**DOI:** 10.3389/fnsyn.2021.798225

**Published:** 2022-01-05

**Authors:** Joseph A. Szule

**Affiliations:** Department of Veterinary Pathobiology, Texas A&M University, College Station, TX, United States

**Keywords:** synapse, neuromuscular junction, active zone, active zone material, neurotransmitter secretion, synaptic vesicle, vesicle trafficking, electron tomography

## Abstract

This report integrates knowledge of *in situ* macromolecular structures and synaptic protein biochemistry to propose a unified hypothesis for the regulation of certain vesicle trafficking events (i.e., docking, priming, Ca^2+^-triggering, and membrane fusion) that lead to neurotransmitter secretion from specialized “active zones” of presynaptic axon terminals. Advancements in electron tomography, to image tissue sections in 3D at nanometer scale resolution, have led to structural characterizations of a network of different classes of macromolecules at the active zone, called “Active Zone Material’. At frog neuromuscular junctions, the classes of Active Zone Material macromolecules “top-masts”, “booms”, “spars”, “ribs” and “pins” direct synaptic vesicle docking while “pins”, “ribs” and “pegs” regulate priming to influence Ca^2+^-triggering and membrane fusion. Other classes, “beams”, “steps”, “masts”, and “synaptic vesicle luminal filaments’ likely help organize and maintain the structural integrity of active zones. Extensive studies on the biochemistry that regulates secretion have led to comprehensive characterizations of the many conserved proteins universally involved in these trafficking events. Here, a hypothesis including a partial proteomic atlas of Active Zone Material is presented which considers the common roles, binding partners, physical features/structure, and relative positioning in the axon terminal of both the proteins and classes of macromolecules involved in the vesicle trafficking events. The hypothesis designates voltage-gated Ca^2+^ channels and Ca^2+^-gated K^+^ channels to ribs and pegs that are connected to macromolecules that span the presynaptic membrane at the active zone. SNARE proteins (Syntaxin, SNAP25, and Synaptobrevin), SNARE-interacting proteins Synaptotagmin, Munc13, Munc18, Complexin, and NSF are designated to ribs and/or pins. Rab3A and Rabphillin-3A are designated to top-masts and/or booms and/or spars. RIM, Bassoon, and Piccolo are designated to beams, steps, masts, ribs, spars, booms, and top-masts. Spectrin is designated to beams. Lastly, the luminal portions of SV2 are thought to form the bulk of the observed synaptic vesicle luminal filaments. The goal here is to help direct future studies that aim to bridge Active Zone Material structure, biochemistry, and function to ultimately determine how it regulates the trafficking events *in vivo* that lead to neurotransmitter secretion.

## Introduction

At chemical synapses, the electrical activity of neuronal axon terminals increases the probability that neurotransmitter molecules in synaptic vesicles (SVs) will be secreted from specialized regions along the presynaptic plasma membrane (PM) called active zones (Katz, 1969; Couteaux and Pecot-Dechavassine, 1970). Prior to secretion, SVs undergo several transient trafficking events (i.e., “docking”, “priming”, “Ca^2+^-triggering” and “membrane fusion”) at active zones that are each necessary for secretion to occur. However, the definition and criteria of these trafficking events are often dependent on the experimental approach, and this has led to many variations of the morphological and biochemical criteria used to define each step (Slater, 2015). For the purpose of this report, docking is described as the directed movement of an SV towards the PM at the active zone, where the SV membrane will be held in direct contact with the PM to become docked. Once an SV is docked, priming influences the probability that the membranes will fuse, as only a small subset of the docked SVs will be triggered to secrete their contents when an electrical impulse arrives (Katz and Miledi, 1979; Heuser and Reese, 1981). Ca^2+^-triggering occurs when Ca^2+^ ions bind specific SV proteins at sufficient concentrations to increase the probability that a docked SV and the PM will undergo membrane fusion, where the two distinct lipid bilayers from each membrane will undergo rearrangements and form a pore that is continuous from the vesicle lumen to the extracellular synaptic cleft (Chernomordik et al., 1995). It is important to note that disruption in the molecular mechanisms of any of these events, either through genetic mutations or pharmacological intervention, will also disrupt the end result of neurotransmitter secretion.

Based on techniques that involve transmission electron microscopy of either tissue sections or freeze-fracture replicas, active zones in presynaptic terminals are generally characterized by the presence of docked SVs held at the PM, a network of macromolecules attached to both the docked SV membranes and the PM called active zone material (AZM), and many large macromolecules that span the PM (Palade, 1954; Palay, 1954; Couteaux and Pecot-Dechavassine, 1970; Heuser et al., 1974, 1979; Propst and Ko, 1987; Harlow et al., 2001). Electron tomography has been used to characterize the fine structure of AZM macromolecules in 3D at nanometer scale resolution *in situ* to provide insights into their direct roles in these trafficking events (Harlow et al., 2001, 2013; Ress et al., 2004; Nagwaney et al., 2009; Fernandez-Busnadiego et al., 2010; Stigloher et al., 2011; Szule et al., 2012, 2015; Matkovic et al., 2013; Imig et al., 2014; Perkins et al., 2015; Cole et al., 2016; Jung et al., 2016, 2018).

Extensive biochemistry studies have also led to a comprehensive characterization of the many conserved protein families universally involved in these trafficking events [reviewed by Rizo and Rosenmund (2008), Sudhof and Rizo (2011), Rizo and Sudhof (2012), and Rizo and Xu (2015)]. Each trafficking event requires specific biochemical interactions between proteins of SVs, AZM macromolecules, and the PM to proceed (Takamori et al., 2006; Südhof and Rothman, 2009; Südhof, 2012, 2013; Snead and Eliezer, 2019), although the mechanistic details of each event are under considerable debate (Hanson et al., 1997; Jahn and Sudhof, 1999; Klenchin and Martin, 2000; Price et al., 2000; Jahn et al., 2003; Szule and Coorssen, 2003; Han et al., 2004; Südhof, 2004; Jackson and Chapman, 2008; Neher and Sakaba, 2008; Chua et al., 2010; Gundersen and Umbach, 2013; Szule et al., 2015). A hypothesized proteomic atlas will be provided here to describe how these conserved proteins are thought to be assembled and function in their AZM macromolecular complexes *in situ* to regulate SV docking, priming, Ca^2+^-triggering, and membrane fusion that ultimately control the regulation of triggered neurotransmitter secretion.

The basic mechanisms for the events that lead to triggered secretion are thought to be conserved across neuron-types due to the homology of the proteins involved, the consistent presence of docked SVs connected to AZM at various active zones, and the universality of Ca^2+^ as the trigger for neurotransmitter secretion (Südhof, 2012; Ackermann et al., 2015). However, there are also well-described differences in protein isoforms and the architecture of AZM which are likely to accommodate synapse-specific physiologies (Palade, 1954; Palay, 1954; Gray, 1963; Zhai and Bellen, 2004; Nagwaney et al., 2009; Ehmann et al., 2014; Ackermann et al., 2015; Slater, 2015). The frog NMJ is a historically established model system of chemical synaptic transmission (Bennett, 1999; Homan and Meriney, 2018); its physiology is well understood (Fatt and Katz, 1952; Kuffler and Vaughan Williams, 1953; Katz and Miledi, 1967, 1979), the organization of axon terminals is known (Couteaux and Pecot-Dechavassine, 1970; McMahan et al., 1972; Heuser et al., 1974, 1979; Ceccarelli and Hurlbut, 1980; Slater, 2003, 2015; Rizzoli and Betz, 2004), and the molecular architecture of its AZM has been quantitatively characterized in 3D by electron tomography at rest and while undergoing SV docking, priming and membrane fusion (Harlow et al., 2001, 2013; Ress et al., 2004; Szule et al., 2012, 2015; Jung et al., 2016). It should be noted that potential artifacts caused by aldehyde fixation and heavy metal staining were addressed using high-pressure freezing and freeze-substitution methods. It was determined that there were no significant differences in the positions and dimensions of the AZM macromolecules (Jung et al., 2016), however, future studies using cryoelectron tomography without the use of heavy metal stains may help refine their unstained dimensions. Thus, AZM at frog NMJs will be used here as a model system to link various conserved proteins involved in neurotransmitter secretion to the macromolecules involved in SV docking, priming, Ca^2+^-triggering, and membrane fusion.

## Overview of Active Zones at Frog Neuromuscular Junctions

**Figure 1:**
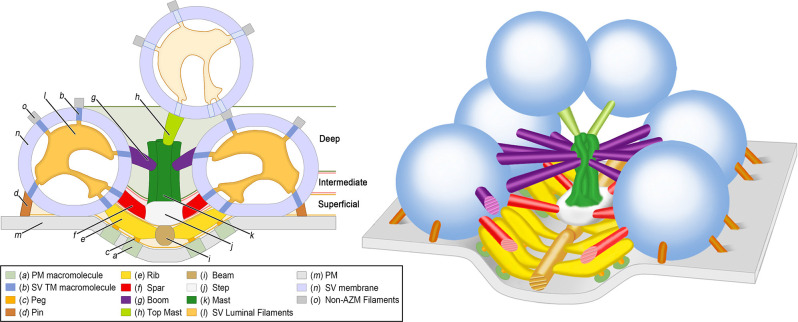
Organization of AZM and SVs at an active zone of a frog NMJ. 2D (Left) and 3D (Right) schematic diagrams derived from electron tomography analysis of active zones showing the positions, dimensions, and connectivity of AZM macromolecules to SV membranes and the PM (see legend for color codes). Docked SVs are in direct contact with the PM whereas undocked SVs are not. The SV luminal filaments and SV transmembrane (TM) macromolecules are ghosted in the undocked SV because their orientations and spatial relationships to AZM and non-AZM macromolecules have not been observed directly. The AZM band is ~1 mm long and is composed of 5–10 repeats of the unit shown in the 3D schematic diagram on the right. Adapted from Harlow et al. (2001); Szule et al. (2012); and Harlow et al. (2013). NMJ, Neuromuscular junction; AZM, Active zone material; PM, Presynaptic membrane; SV, Synaptic vesicle.

Active zones in a motor neuron axon terminal at frog NMJs are situated immediately across the synaptic cleft from a junctional fold in the post-synaptic muscle cell membrane (Couteaux and Pecot-Dechavassine, 1970). The main body of AZM is a band that is ~1 μm long, ~50 nm wide, and extending ~75 nm into the cytoplasm. It is flanked on each side by ~10–20 docked SVs, a small portion of which (1–3%) will secrete their neurotransmitter cargo when the axon terminal is stimulated by an electrical impulse (Couteaux and Pecot-Dechavassine, 1970; Heuser et al., 1974). Further, the macromolecules that span the PM at the active zone are organized in a parallel double row array (Heuser et al., 1974, 1979; Ceccarelli et al., 1979a, b; Fesce et al., 1980; Stanley et al., 2003). Transmission electron tomography has shown that AZM is highly ordered. It is composed of morphologically distinct classes of macromolecules that are categorized based on their relative positions, their dimensions, and their connectivity to the SVs, the PM, and the other AZM macromolecules (Figure 1; Table 1; Harlow et al., 2001, 2013; Szule et al., 2012). Although AZM macromolecules are defined as morphologically distinct structures, their extensive connection to each other, the SVs (both docked and undocked) and the PM make it likely that different domains of individual proteins are components of more than one AZM macromolecule. Further, the dimensions of each AZM macromolecule are also sufficient to accommodate multiple proteins. These results have been described (Harlow et al., 2001, 2013; Ress et al., 2004; Szule et al., 2012; Jung et al., 2016), and reviewed (Szule et al., 2015), but a brief description will be provided here.

**Table 1:** Dimensions of active zone material (AZM) macromolecules.

	Mean ± S.D. nm *(n)*		
AZM macromolecule	Length	Diameter	Reference
Ribs	~28	~9	Szule et al. (2012)
Proximal portion of ribs	~17		Jung et al. (2016)
Pegs	≤7		Harlow et al. (2001)
Pins	~13	~5	Jung et al. (2016)
Spars	~18	~7	Szule et al. (2012)
Booms	~16	~7	Szule et al. (2012)
Top-Masts	~25	~7	Szule et al. (2012)
Steps	~28 × ~22	~14	Szule et al. (2012)
Mast bundle of filaments	~32	~22	Szule et al. (2012)
Mast filaments	~32	~9	Szule et al. (2012)
Beams	~75	~11	Harlow et al. (2001) and Szule et al. (2012)

The superficial layer of AZM, ≤15 nm from the PM, consists of beams, ribs, pegs, and pins (Figure 1; Table 1). Beams are situated adjacent to the PM and their long axis runs parallel to the long axis of the active zone. Ribs, which are also situated adjacent to the PM but perpendicular to beams, connect to beams and docked SV membrane. Pegs are short filaments that connect ribs to the PM-spanning macromolecules that are arranged in the distinguishing parallel double row array (Heuser et al., 1974; Pumplin et al., 1981; Harlow et al., 2001). Pins connect to the SV membrane and the PM and are situated around the region of contact between these membranes.

The intermediate layer of AZM, ~15–30 nm from the PM, consists of steps and spars (Figure 1; Table 1). Steps are situated periodically along the midline of the AZM band deeper into the cytoplasm compared to beams. Spars connect to steps near the midline of the band and to docked SVs at the periphery of the band.

The deep layer of AZM, ~30–75 nm from the PM, consists of masts, booms, and top-masts (Figure 1; Table 1). Masts extend from the steps perpendicular to the plane of the PM and consist of four to nine thinner fibers. Booms connect to masts and to docked SVs. Top-masts connect to masts and to the membrane of nearby undocked SVs. Booms and top-masts have a comparable mean diameter thickness, both classes of structures connect to the masts in similar positions, and top-masts occur in variable angular orientations. Thus, it is conceivable that booms and top-masts are the same macromolecular complexes and that their differences described here are based upon whether the SV that they connect at a resting active zone is docked or undocked.

SV luminal filaments can be visualized by transmission electron microscopy in frog NMJs that had been fixed and stained by high pressure freezing and freeze-substitution, but not when aldehyde fixed and heavy metal stained at room temperature (Harlow et al., 2013). Interestingly, cryoelectron tomography on cultured CNS neurons revealed that the lumen of some SVs also contained filamentous material (Schrod et al., 2018). The SV luminal filaments at frog NMJs, found in both docked and undocked SVs, occupies ~10% of the luminal volume and forms a chiral structure that radiates from the center of the lumen to provide each SV a distinguishable orientation (Harlow et al., 2013; Figure 1). The filaments connect to the luminal surface of an SV membrane by ~25 nub connection sites, which are also stereotypically arranged, and link to the different classes of AZM and non-AZM macromolecules by transmembrane macromolecules. The many different SV transmembrane proteins, that have variously sized luminal domains (Takamori et al., 2006; Burré and Volknandt, 2007), have been proposed to link in the SV lumen in a specified configuration so as to predefine where the proteins of the AZM macromolecules connect to the cytosolic surface of the SV membrane, i.e., the so-called AZM-binding domain (Harlow et al., 2013).

### SV Trafficking Events at Active Zones of Frog Neuromuscular Junctions

#### SV Docking

**Figure 2:**
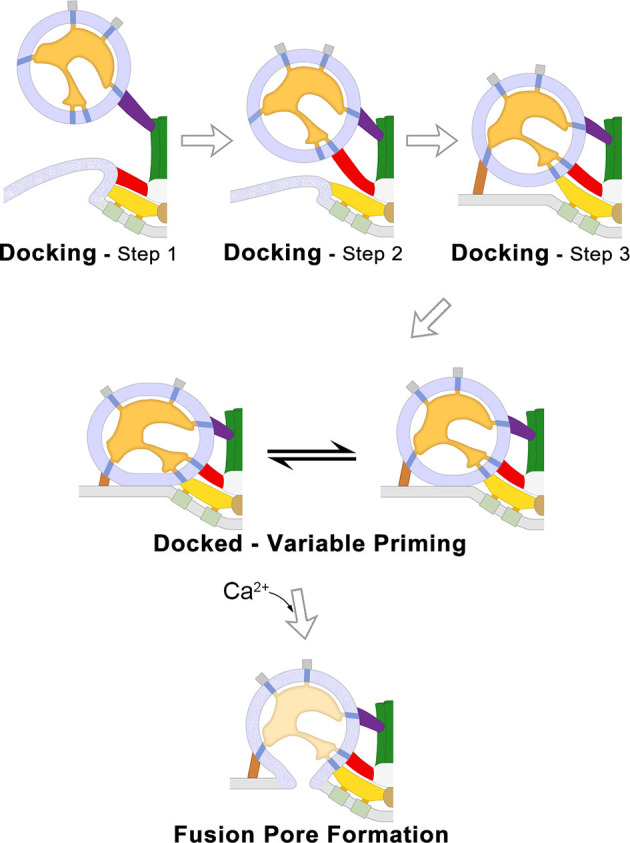
SV traffickingevents that lead to neurotransmitter secretion at frog NMJs. 2Dschematic diagrams of the SV trafficking events at frog NMJs derivedfrom electrically stimulated terminals and analyzed by electrontomography. The three steps of SV docking are characterized by the distance between the undocked SV to the PM and their connections to specific AZM macromolecules (Top). After an SV is docked it undergoes priming which determines the probability that it will fuse when an electrical impulse arrives; the variable priming model states that priming is regulated by forces exerted by AZM macromolecules, which are variable and in dynamic equilibrium, to destabilize the SV membrane–PM contact site and change the positioning of voltage-gated Ca^2+^-channels in relation to the Ca^2+^-sensor protein embedded in the docked SV membrane (Middle). When sufficient concentrations of cytosolic Ca^2+^ bind the sensor protein Synaptotagmin, the SV membrane and PM undergo lipidic membrane fusion to form a pore through which neurotransmitter molecules are secreted to the synaptic cleft (Bottom). Adapted from Szule et al. (2012) and Jung et al. (2016).

To test the involvement of AZM in the SV trafficking events at frog NMJs, the axon terminals of motor neurons were chemically fixed during high-frequency electrical stimulation. Fixation-stabilized “snapshots” of undocked SVs were captured during their transition to becoming docked while interacting with the different classes of AZM macromolecules to discern the morphological interactions and steps during SV docking (Figure 2-Top; Szule et al., 2012). During step 1 of docking, the full complement of ~7 booms stably connects with the undocked SV when it is 30–40 nm from the PM which may function to draw the SV to the PM and/or orient the SV so that it is able to interact with other AZM macromolecules. During step 2 of docking, the SV stably connects with the full complement of ~2 spars when it is 17–24 nm from the PM, in addition to the previous connections with booms, which may also draw the SV to the PM and stabilize its orientation to facilitate interaction with other AZM macromolecules. During step 3 of docking, undocked SVs interact with the full complement of ~4 ribs and ~4 pins when it is less than 16 nm from the PM, in addition to the previous connections with spars and booms, which may function to fine-tune the alignment of the SV prior to becoming docked on a predefined, specialized position of the PM. Once the SV has interacted with the full complement of AZM macromolecules, force is likely applied between the SV membrane and PM to overcome the repulsive and hydration forces that are present upon their close apposition (Rand and Parsegian, 1989), bringing the SV membrane into direct contact with the PM where it becomes docked.

#### SV Priming

Electron tomography was also used to study the structural role of AZM macromolecules during docked SV priming at active zones from frog NMJs. It was determined that there are correlations between several structural parameters with the probability that the SV will fuse when the terminal is electrically stimulated, including the area of SV-PM contact, the length of ribs and pins, and the positions of pegs (Jung et al., 2016; Figure 2-Middle). Further, electron microscopy of frog NMJ freeze-fracture replicas led to the conclusion that the positions of transmembrane macromolecules, and consequently their associated pegs, are dynamic during exocytosis (Stanley et al., 2003). Thus, it was hypothesized that priming for each docked SV is continuously changing (Figure 2-Middle). The area of SV-PM contact and the position of the pegs and their associated PM-spanning macromolecules is thought to be due to force being applied by the shortening ribs and pins, which resulted in increased membrane destabilization and movement of the putative voltage-gated Ca^2+^-channels closer to the Ca^2+^-sensor protein at the SV-PM interface (Jung et al., 2016).

#### Ca^2+^-Triggering and Membrane Fusion

Ca^2+^ ions that enter the axon terminal through voltage-gated Ca^2+^-channels when an electrical impulse depolarizes the PM bind to Ca^2+^-sensor proteins embedded on the SV membrane. At sufficient concentrations, Ca^2+^ binding to the sensor protein changes its interactions with the PM (Chapman and Davis, 1998; Hui et al., 2009; Paddock et al., 2011; Bowers and Reist, 2020). These changes are thought to overcome an energy barrier and initiate membrane lipid rearrangements that ultimately result in the formation of a fusion pore between the SV membrane and PM, as described by the stalk-pore hypothesis (Chernomordik et al., 1995; Kozlov et al., 2010). At frog NMJs, fused SVs undergo full fusion (Figure 2-Bottom) and the SV membrane then flattens into the PM and moves to a lateral position where it is endocytosed (Heuser and Reese, 1973). There is little evidence for “Kiss-and-Run” fusion at frog NMJs (Rizzoli and Jahn, 2007), as compared to other synapses throughout the nervous system (Alabi and Tsien, 2013), and the proteins involved in endocytosis and SV recycling are beyond the scope of this report, but see Doherty and McMahon (2009). SVs that had fused and vacated the docking sites are replaced at the active zone by a nearby undocked SV from the recycling pool (Rizzoli and Betz, 2005), presumably by one that is connected to a top-mast (Szule et al., 2012).

The AZM macromolecules that connect to SV membranes are likely formed through specific interactions between proteins of a base AZM complex attached to the PM and proteins bound to SV membranes. The functions and interacting domains of various proteins that contribute to AZM structures at different synapses have been comprehensively reviewed elsewhere (Südhof, 2004; Schoch and Gundelfinger, 2006; Takamori et al., 2006; Rizo and Rosenmund, 2008; Chua et al., 2010), although it is important to note that not all of these proteins have been specifically identified at active zones of frog NMJs. Here, a hypothesis is presented that relates the contributions of several proteins that have been implicated in AZM regulated vesicle trafficking events to the different classes of AZM macromolecules. The hypothesis considers the common roles, binding partners, physical features/structure, and relative positioning in the axon terminal of both the proteins and classes of macromolecules involved in the vesicle trafficking events. The structures of several proteins listed below have been determined by x-ray crystallography, single particle cryo-electron microscopy, or NMR spectroscopy in solution and deposited in the Protein Data Bank (PDB^1^). The length, width, and depth of several protein structures, at their greatest distance in each dimension, were measured here unless otherwise stated using the “Measurements” tool from the Mol* Viewer software package (Sehnal et al., 2021).

## Hypothesis: Proteins That Constitute “Active Zone Material” Macromolecules

### Cation Channels

*N-type Ca^2+^-channels* (Ca_V_2.2; Catterall, 2000–2013), the prominent type of voltage-gated Ca^2+^-channels present at active zones of frog NMJs (Robitaille et al., 1990; Cohen et al., 1991), allow the influx of Ca^2+^ into the cytosol in response to membrane depolarization to trigger membrane fusion. The channel consists of the α_1B_ pore-forming subunit, β subunit, and α_2_/β_1_ subunits. α_1B_ also has a large cytoplasmic domain that includes the 87 amino acid “synprint” region in the II-III linker that interacts with a cytosolic region of the SNARE protein syntaxin and influences channel gating (Sheng et al., 1994; Bezprozvanny et al., 1995; Jarvis et al., 2002). The single particle cryo-electron microscopy-derived structure of the α_1B_ subunit [PDB accession code: 7MIY (Gao et al., 2021)] has an expected diameter in the plane of the PM of ~11 nm (Table 2).

**Table 2:** Hypothesis of protein contributions to the classes of AZM macromolecules.

Protein	Putative function	PDB accession	Dimensions, nm (LxWxD)	AZM structure
N-type Ca^2+^-channel	Cation regulation	7MIY	11 × 11 × 22	Pegs, Ribs
Ca^2+^-gated K^+^ channels	Cation regulation	1LNQ	13 × 13 × 13	Pegs, Ribs
Syntaxin	Late stage of secretion	1N7S	10 × 1 × 1	Ribs/Pegs, Pins
SNAP25	Late stage of secretion	1N7S	12 × 2 × 2	Ribs, Pins
Synaptobrevin	Late stage of secretion	1N7S	9 × 1 × 1	Ribs, Pins
SNARE complex	Late stage of secretion	1N7S	12 × 3 × 3	Ribs, Pins
Synaptotagmin	Ca^2+^-sensor for secretion	5CCG	8 × 5 × 5	Ribs, Pins
Munc13	SNARE complex regulation			Ribs, pins
Munc18	SNARE complex regulation	6LPC	8 × 8 × 5	Ribs
Complexin	SNARE complex regulation	3RK3	8 × 1 × 1	Ribs
NSF	SNARE complex regulation	3J95	13 × 13 × 9	Ribs
Rab3A	SV Tethering and Docking	1ZBD	5 × 4 × 3	Top-Masts, Booms, Spars
Rabphilin-3A	SV Tethering and Docking	1ZBD	8 × 3 × 2	Top-Masts, Booms, Spars
Rab3A-Rabphilin-3A	SV Tethering and Docking	1ZBD	8 × 5 × 3	Top-Masts, Booms, Spars
RIM	Scaffolding			Beams, Steps, Masts, Ribs, Spars, Booms, Top-Masts
Bassoon/piccolo	Scaffolding		80 × 10 × 10	Beams, Steps, Masts, Ribs, Spars, Booms, Top-Masts
Spectrin	Scaffolding			Beams
SV2	Vesicle Scaffolding			SV luminal filaments

*Ca^2+^-gated K^+^-channels* found at active zones of frog NMJs regulate the efflux of K^+^ to repolarize the membrane potential in preparation for subsequent rounds of triggered secretion (Robitaille and Charlton, 1992; Robitaille et al., 1993a,b). From the x-ray diffraction-derived structure of Ca^2+^-gated K^+^-channels [PDB accession code: 1LNQ (Jiang et al., 2002)], the diameter in the plane of the PM is expected to range from ~8 to 13 nm (Table 2).

The freeze-fracture replicas of macromolecules that span the PM at active zones each have diameters that range from 9 to 13 nm (Fesce et al., 1980) which includes a thin coating of platinum/carbon.

Thus, based on the requirement for cation flux through the PM at active zones to mediate neurotransmitter secretion and similarities in the dimensions of the cation channels and the PM-spanning macromolecules observed by freeze-fracture techniques at active zones of frog NMJs, it is hypothesized that the N-type Ca^2+^-channels and Ca^2+^-gated K^+^ channels are included in the macromolecules that span the PM, and their large cytoplasmic domains, including the synprint region, are included in pegs and ribs (Table 2). However, the specific composition and arrangements of the channel types in relation to each other and the docked SVs are under investigation. Through computational modeling, it has been estimated that on average two, but as few as one, active N-type Ca^2+^-channels associated with a docked SV are required to trigger membrane fusion (Dittrich et al., 2013; Homan et al., 2018). Further, it has also been proposed that the N-type Ca^2+^-channels are included in the rows that are proximal to the docked SVs (Jung et al., 2016).

### The SNARE Proteins

Syntaxin, SNAP25 with Synaptobrevin together referred to as SNARE proteins (Soluble NSF-Attachment Protein Receptor) assemble to form the SNARE complex, and several models have implicated the complex as essential for membrane fusion (Sollner et al., 1993b; Weber et al., 1998; Melia et al., 2002; Han et al., 2004; Südhof and Rothman, 2009; Jackson, 2010; Karatekin et al., 2010). Additionally, other models suggest the roles of the SNARE complex to be upstream to membrane fusion, such as during docking and priming (Coorssen et al., 1998; Tahara et al., 1998; Price et al., 2000; Harlow et al., 2001, 2013; Szule and Coorssen, 2003, 2004; Szule et al., 2003, 2012; Gundersen and Umbach, 2013; Imig et al., 2014; Meriney et al., 2014; Jung et al., 2016).

*Syntaxin* has a transmembrane domain that spans the PM associated with N-type Ca^2+^ channels through the synprint domain (Bennett et al., 1992b; Sheng et al., 1994; Bezprozvanny et al., 1995; Rettig et al., 1997; Jarvis et al., 2002). *SNAP25* has been post-translationally modified with palmitoyl lipid moieties so that it associates with and is anchored in the hydrophobic core of the PM (Veit et al., 1996). *Synaptobrevin*, also referred to as VAMP (Vesicle-Associated Membrane Protein), has a transmembrane domain that is integral to the SV membrane. The PM-associated proteins Syntaxin and SNAP25 interact with the SV membrane-associated protein Synaptobrevin to form a SNARE core complex through associations of their so-called SNARE coiled-coil domains. The SNARE core complex forms a four-helix bundle with contribution of one coiled-coil domain (i.e., a characteristic 65 amino acid stretch) from Syntaxin, two from SNAP25, and one from Synaptobrevin [reviewed by Sudhof and Rizo (2011) and Rizo (2018)]. Syntaxin1, SNAP25, and VAMP2 are neuronal isoforms, and are present at active zones of frog NMJs (Boudier et al., 1996). From x-ray crystallography [PDB accession code: 1SFC (Sutton et al., 1998); PDB accession code: 1N7S (Ernst and Brunger, 2003)] and cryo-electron microscopy [PDB accession code: 6MTI (Grushin et al., 2019)], the SNARE core complex is ~12 nm in length and ~3 nm in diameter (Table 2). Further, it has been estimated that multiple SNARE core complexes, at least three in cultured PC12 cells, are associated with each docked vesicle for biological secretion to proceed (Hua and Scheller, 2001). Once assembled, the SNARE core complex is variable in length and it is proposed to zipper, and effectively shorten, to exert force between the two opposing membranes (Weber et al., 1998; Melia et al., 2002).

Pins and ribs/pegs are connected to both the PM and SV membranes, in agreement with the assignment of the SNARE core complex to these AZM macromolecules. The physical dimensions of the SNARE core complex can be accommodated by pins (~13 nm in length and 5 nm in diameter; Table 1), and the proximal portion of ribs between the peg proximal to the docked SV and the SV membrane (~17 nm in length and ~9 nm in diameter; Table 1). It was concluded that pins and proximal portions of ribs change the length to exert variable amounts of force between the SV membrane and PM during the final stage of docking and during priming, as would be expected if SNARE complexes were included in these structures (Szule et al., 2012; Jung et al., 2016). However, further scrutiny suggests that the proximal portions of ribs are associated with pegs that are associated with large macromolecules transmembrane to the PM likely to include voltage-gated Ca^2+^-channels (Harlow et al., 2001), whereas pins have not been documented to be associated with any such large macromolecules that span the PM. Therefore, while it is plausible that SNARE core complexes are components of both pins and ribs, either only a subset of SNARE core complexes associate with the voltage-gated Ca^2+^-channels or they are only components of the proximal portions of ribs (Table 2).

### Synaptotagmin

*Synaptotagmin* is the putative Ca^2+^-sensor protein to trigger neurotransmitter secretion (Perin et al., 1990; Mackler et al., 2002). There are 17 isoforms of Synaptotagmin that impart different Ca^2+^-sensitive cellular functions, and Synaptotagmin 1 has been shown to be present at active zones of frog NMJs (Boudier et al., 1999). For a review of the different isoforms of Synaptotagmin, see Südhof (2002) and Wolfes and Dean (2020). Synaptotagmin has a short SV luminal domain, a transmembrane domain that is integral to the SV membrane, SNARE-interacting domains, and two cytoplasmic Ca^2+^-binding C_2_ domains (C_2_A and C_2_B) that interact with the plasma membrane (Rizo and Sudhof, 1998; Groffen et al., 2010; Grushin et al., 2019). The SNARE interacting domain consists of multiple binding sites between the C_2_ domains and the SNARE core complex, with the primary interface between C_2_B and the Syntaxin-SNAP25 complex suggested to be involved triggered secretion that occurs at motoneuron active zones (Zhou et al., 2015). Ca^2+^-binding to Synaptotagmin changes its interactions with the plasma membrane by masking repulsive electrostatic charges and inducing insertion of hydrophobic residues of the C_2_ domains into the hydrophobic region of lipid bilayers to lower the energy barrier of membrane fusion (Chernomordik et al., 1995; Chapman and Davis, 1998; Hui et al., 2009; Kozlov et al., 2010; Paddock et al., 2011; Bowers and Reist, 2020). Further, in the absence of Ca^2+^-binding, Synaptotagin 1 and 2 form oligomeric rings that are 20–40 nm in diameter (Wang et al., 2014; Zanetti et al., 2016).

The x-ray crystallography-derived structure of Synaptotagmin 2 [PDB accession code: 5CCG (Zhou et al., 2015)] is ~8 nm × ~5 nm × ~5 nm (Table 2).

Pins and ribs/pegs are connected to both the PM and SV membranes, and their ~8 connections form a ring around the SV-PM contact area that has been measured to have an average diameter of 20–25 nm (Szule et al., 2012; Jung et al., 2016). This arrangement agrees with the assignment of Synaptotagmin in the proximal portions of ribs and pins. Synaptotagmin is transmembrane to the SV membrane and the SNARE interacting domains are situated with, and bound to, SNARE core complexes, which are proposed to be included in the proximal portions of ribs and pins. The C_2_ domains interact with the PM, and the connection sites of ribs/pegs and pins with the PM are closest to the SV membrane—PM contact site when the SV is most primed. And, the rings formed by oligomers of Synaptotagmin are similar in dimension to the rings formed by ribs/pegs and pins around the SV membrane—PM contact site. Further, these AZM macromolecules are of sufficient size to accommodate Synaptotagmin. Thus, it is proposed that Synaptotagmin is included in the proximal portions of ribs and/or pins (Table 2).

### SNARE Auxiliary Proteins

*Munc13* is a large protein (~200 kD) that is thought to be involved in SV priming (Augustin et al., 1999), operating through interactions with membrane lipids including diacylglycerols (Basu et al., 2007), and SNARE proteins (Betz et al., 1997). It has been proposed that Munc13 interactions with Syntaxin regulate the associations between Syntaxin and SNAP25, thereby providing an acceptor complex for Synaptobrevin (Guan et al., 2008). There are several domains of Munc13 including C_2_A domain, CaMb (Calmodulin-binding sequence), C1 (membrane diacylglycerol lipid-binding), C_2_B, MUN, and C_2_C, however, the structure-function relationship of several of these domains remain unclear. Further, the structure of Munc13 in its entirety has not yet been solved, but rather only certain domains have been characterized including a fraction of the MUN-CD domain which has been proposed to be structurally similar to other membrane tethering domains (Li et al., 2011). A fragment of Munc13, that includes the C1, C_2_B, and MUN domains, is elongated and reported to be 19.5 nm in length (Xu et al., 2017). It has also been proposed that the C1-C_2_B-MUN-C_2_C domains bridge the SV membrane to the PM (Quade et al., 2019).

*Munc18* is a member of the Sec1/Munc18 (SM) family of proteins that are conserved and critical for different types of membrane trafficking (Carr and Rizo, 2010). Munc18 is thought to be involved in SV priming by controlling the formation of the SNARE complex through direct interactions with the closed conformation of Syntaxin, thereby stabilizing it and hindering the assembly of the SNARE core complex (Dulubova et al., 1999, 2003; Rizo and Sudhof, 2002; Burkhardt et al., 2008; Gerber et al., 2008). It has further been proposed that Munc13 and Munc18 cooperatively chaperone SNARE complex assembly prior to zippering (Shu et al., 2020). The x-ray crystallography-derived structure of Munc18 [PDB accession code: 6LPC (Wang et al., 2020)] is ~8 nm × ~8 nm × ~5 nm (Table 2).

*Complexin* is a cytosolic protein that interacts with the SNARE complex (Chen et al., 2002) at a position that also binds Synaptotagmin (Tang et al., 2006) and has been proposed to act as a clamp that inhibits membrane fusion by inhibiting the complete zippering of the SNARE complex (Giraudo et al., 2006). In this model, Complexin is dislodged from the SNARE complex in a Ca^2+^-dependent manner to allow membrane fusion to proceed. In an alternate and contradictory model, Complexin has been proposed to facilitate secretion because deletion of Complexin results in reduced Ca^2+^-triggered neurotransmitter release in synapses of the mouse central nervous system (Xue et al., 2008). Regardless of its physiological role in secretion, it is established that Complexin interacts with the SNARE complex. The x-ray crystallography-derived structure of Complexin [PDB accession code: 3RK3 (Kummel et al., 2011)] is ~8 nm × ~1 nm × ~1 nm (Table 2).

*NSF* (N-ethylmaleimide Sensitive Factor) is an ATPase that, together with SNAP (Soluble NSF Attachment Protein), has been proposed to disassemble the SNARE complex after membrane fusion has occurred so that Syntaxin and SNAP25 can interact with Synaptobrevin of an incoming SV and form a new SNARE complex (Sollner et al., 1993a). NSF binds a subcomplex of SNAP protein and cis-SNARE complex, i.e., Syntaxin, SNAP25, and Synaptobrevin are anchored in the same membrane after fusion, to form a so-called 20S super-complex. ATP hydrolysis initiates NSF to exert torque to unwind the highly stable four-helix SNARE complex bundle (Zhao et al., 2015), and is present in active zones of frog NMJs (Boudier et al., 1996). The single particle cryo-electron microscopy-derived structure of NSF [PDB accession code: 3J95 (Zhao et al., 2015)] is ~13 nm × ~13 nm × ~9 nm (Table 2).

Munc13, Munc18, Complexin, NSF each directly associate with the SNARE proteins, that are designated above to be included in the ribs and/or pins. The diameter of the SNARE complex, Munc18, and Complexin (not including Munc13 because its dimensions are unknown) is 9 nm if they were bound together, and less if their binding positions with the SNARE complex were staggered, which can be accommodated by the average diameter of the ribs (9 nm; Table 1). Further, the average full length of ribs (29 nm; Table 1) or just the proximal portion of ribs (17 nm; Table 1) are sufficient to accommodate this complex if their binding to the SNARE complex were staggered. However, it is unlikely that the diameter of the pins (5 nm; Table 1) can accommodate this large complex but it is plausible that pins accommodate fewer proteins bound with the SNARE complex or possibly the Munc13 bridge between the SV membrane and the PM. It is also established that NSF binds the cis-SNARE complex, i.e., they are anchored in the same membrane after the SV and PM had fused, which presents the possibility that it is a component of AZM at rest while the SNARE complex is in a trans configuration, i.e., Syntaxin and SNAP25 anchored in the PM and Synaptobrevin anchored in the SV membrane. However, it is also plausible that NSF is recruited from the cytosol by the presence of the cis-SNARE complex after membrane fusion had occurred. Thus, it is hypothesized here that Munc13 is localized to ribs and/or pins, Munc18, and Complexin, are localized to ribs, and NSF is either a component of ribs at rest or binds ribs and/or pins after membrane fusion had occurred (Table 2).

### Rab3A and Rabphilin-3A

Rab proteins constitute a large family of low molecular mass GTP-binding proteins that are involved in multiple stages of membrane trafficking throughout the cell (Grosshans et al., 2006; Hutagalung and Novick, 2011). They interact with effectors preferentially while in a GTP-bound state through a Switch domain (Pfeffer, 2005). Rab3 proteins are a sub-family that associate with SVs during the late stages of membrane trafficking (Matteoli et al., 1991; Geppert et al., 1997), however, there are multiple isoforms of Rab3 which may have multiple functions, making interpretations of knock-out and over-expression studies difficult (Schluter et al., 2002). *Rab3A* is the most abundant Rab3 protein in the nervous system and in its GTP-bound state translocates from the cytosol to interact with the hydrophobic region of the SV membrane and its effector *Rabphilin-3A* (Stahl et al., 1996). Rabphilin-3A possesses 2 tandem C_2_ domains that bind to SV membranes in a Ca^2+^-dependent manner (Chung et al., 1998). SV redistribution within axon terminals of *C. elegans* and mouse motor-neurons has been demonstrated in Rab3 and Rab3A mutant animals, respectively, with a reduced proportion of SVs at active zones within <50–150 nm of the PM (Nonet et al., 1997; Coleman et al., 2007). It was concluded that Rab3A is not essential for SV fusion with the PM, but rather is required to maintain a normal reserve of SVs during repetitive stimulation by directing them to the active zones (Südhof, 1995; Nonet et al., 1997; Coleman et al., 2007). From the x-ray crystallography-derived structure of Rab3A/Rabphilin-3A complex [PDB accession code: 1ZBD (Ostermeier and Brunger, 1999)] Rab3A is ~5 nm × ~4 nm × ~3 nm, Rabphilin-3A is ~8 nm × ~3 nm × ~2 nm, and the overall Rab3A/Rabphilin-3A complex is ~8 nm × ~5 nm × ~3 nm (Table 2).

Rab3A-Rabphilin-3A are directly involved in the interactions between the AZM and the SV membranes at an active zone. The interactions between AZM and the membranes of undocked and docking SVs involve the top masts, booms, spars, ribs and pins, and the dimensions of each are sufficient to accommodate the inclusion of Rab3A and Rabphilin-3A. However, as outlined above, it is likely that the rib connections to docking SV membranes involve the SNARE proteins to form a SNARE core complex to exert force between the docking SV membrane and the PM to bring them into direct contact. Further, pin-SV membrane connections are not likely to involve Rab3A-Rabphilin-3A interactions because pins are directly involved in step 3 of docking once the SV is 15 nm from the PM and in regulating priming for Ca^2+^-triggered membrane fusion once the SV is docked on the PM (Figure 2; Szule et al., 2012; Jung et al., 2016). Also, the pin-SV interactions are not likely involved in maintaining a normal reserve of undocked SVs during stimulation or affecting the movement/state/positioning of undocked SVs during the early steps of docking when it is >15 nm from the PM. Top-masts are likely to be involved in maintaining a normal reserve of undocked SVs at the active zone during stimulation, and the booms and spars are likely to affect the positioning of undocked SV when they are further than 15 nm from the PM. Therefore, it is hypothesized that Rab3A and Rabphilin-3A are localized to the interface of SV membranes with the top-masts, and/or booms, and/or spars (Table 2).

### AZM Scaffolding Proteins

The AZM consists of several multidomain scaffolding proteins that interact with other proteins enriched at active zones (Schoch and Gundelfinger, 2006; Mittelstaedt et al., 2010).

*RIM* (Rab3-Interacting Molecules) protein is generally thought to be a critical active zone organizer that recruits voltage-gated Ca^2+^-channels and is involved in SV docking and priming (Zarebidaki et al., 2020). There are seven members of the RIM protein family, encoded by four genes, with RIM1α likely involved in neurotransmission. RIM1α has a zinc-finger, PDZ, C_2_A, and C_2_B domains. RIMs have been reported to bind with Rab3A in a GTP-dependent manner (Wang et al., 1997), with Munc13 to form a Rab3-RIM-Munc13 tripartite complex (Dulubova et al., 2005), directly with voltage-gated Ca^2+^-channels (Kiyonaka et al., 2007; Picher et al., 2017), and with other scaffolding proteins such as liprin-α and ELKS (Schoch et al., 2002; Wang et al., 2002; Lu et al., 2005). Although the structure of RIM in its entirety has not yet been solved, the structures of individual domains have been derived by solution NMR or x-ray crystallography. The structures of the zinc-finger domain [PDB accession code: 2A20 (Dulubova et al., 2005)] is ~4 nm × ~3 nm × ~2 nm, the PDZ domain [PDB accession code: 1ZUB (Lu et al., 2005)] is ~4 nm × ~4 nm × ~2 nm, the C_2_A domain [PDB accession code: 2BWQ (Dai et al., 2005)], and the C_2_B domain [PDB accession code: 2Q3X (Guan et al., 2007)] is ~5 nm × ~4 nm × ~3 nm (Table 2).

*Bassoon* and *Piccolo* are scaffolding proteins that are enriched at the synaptic active zone, they share high sequence similarity, and have several similar protein interacting domains (Cases-Langhoff et al., 1996; tom Dieck et al., 1998); reviewed by Gundelfinger et al. (2016). Both Basoon and Piccolo are thought to be vertebrate-specific and have been found at active zones of synapses from both the central and peripheral nervous systems. They are also thought to perform multiple presynaptic functions including assembly of active zones, organization of neurotransmitter release machinery, endocytosis, and synapse maintenance. Bassoon and Piccolo have two zinc-finger, three coiled-coil, PDZ, C_2_A, and C_2_B domains that perform the various functions and bind with other active zone proteins. These proteins include, but are not limited to, Munc13, CAST (CAZ-Associated Structural Protein; an active zone scaffolding protein that is structurally related to ELKS), RIM, and voltage-gated Ca^2+^-channels (Takao-Rikitsu et al., 2004; Wang et al., 2009; Chen et al., 2011; Gundelfinger et al., 2016). Using immunohistochemistry and super-resolution STED microscopy on active zones at mouse NMJs (Nishimune et al., 2016), Bassoon and Piccolo were shown to be localized to AZM in the vicinity of the voltage-gated Ca^2+^-channels. Although the structures of Bassoon and Piccolo in their entirety have not yet been solved, silica modeling has predicted their structures based on x-ray crystallography and solution NMR of the multiple domains (Gundelfinger et al., 2016). Overall, Bassoon and Piccolo have an elongated length of ~80 nm that generally appears filamentous with interspersed globular domains that are estimated to be less than ~10 nm in diameter (Table 2).

*Spectrins* are a family of cytoskeletal proteins separated into α-Spectrins (αI, αII) and β-Spectrins (βI, βII, βIII, βIV, βV), which are each composed of 2 α and 2 β subunits. Spectrins contain a Calponin Homology (CH) domain, SRC Homology 3 (SH3) domain, Pleckstrin Homology (PH) domain, EF hand domain, and spectrin repeats, and there are binding sites for other proteins including ankyrin, actin, synapsin, among others, and membranes containing PIP_2_, phosphatidylserine, and phosphatidylethanolamine lipids (reviewed by Machnicka et al., 2014). Spectrins generally create membrane scaffolds at Golgi, endoplasmic reticulum, and plasma membrane with various functions during cellular trafficking (reviewed by De Matteis and Morrow, 2000). Brain-derived Spectrins have been shown to interact with the presynaptic protein Synapsin I (Sikorski et al., 1991), and β-Spectrin has been shown to interact either directly or indirectly with several active zone proteins including Munc13 in rat brain (Sakaguchi et al., 1998), and voltage-gated Ca^2+^ channels at active zones of the torpedo electric organ synapse which is a modified NMJ (Sunderland et al., 2000). The x-ray crystallography-derived structure of the Spectrin repeat region of β-Spectrin [PDB accession code: 6M3P (Li et al., 2020)] is ~15 nm × ~3 nm × ~2 nm, which link to form elongated filaments.

RIM likely interacts with Rab3A in the top-masts and/or booms and/or spars, Munc13 in the ribs, and voltage-gated Ca^2+^-channels in ribs/pegs, as outlined above. However, for RIM to simultaneously interact with Rab3A, Munc13, and voltage-gated Ca^2+^-channels, it is also likely to be a component of beams, steps, and masts. Bassoon and Piccolo function by inducing the assembly and organization of AZM, they possess several protein-binding domains, and their lengths can extend across the depth of AZM. It is likely that their interactions with voltage-gated Ca^2+^-channels, Munc13 and RIM are localized to pegs, ribs, spars/booms/top-masts. Further, the other scaffolding interactions between Bassoon/Piccolo, RIM, Liprin-α, and CAST/ELKS are likely to occur in the central regions of the AZM (i.e., in the beams, steps, and masts). Spectrin is an elongated filamentous cytoskeletal protein at the interface with the PM, and it possesses several domains that bind other AZM proteins proposed to be at or near the PM including voltage-gated Ca^2+^ channels and Munc13. Beams are also elongated (~75 nm; Table 1) filamentous AZM macromolecules at the interface with the PM that are connected to ~10–12 ribs, which are thought to include voltage-gated Ca^2+^ channels and Munc13. Thus, it is hypothesized here that Spectrin is a component of beams.

### Vesicle Scaffolding

The protein backbone of the luminal filaments is thought to be glycosylated, which forms a carbohydrate matrix that helps bind the soluble content of the SVs (Rahamimoff and Fernandez, 1997; Reigada et al., 2003). It was further hypothesized that the SV luminal filaments organize the locations of AZM macromolecule connections on the external membrane surface and impart the SV with a distinct orientation (Harlow et al., 2013).

*SV2* (Synaptic Vesicle protein 2) is an ~80 kD, highly glycosylated protein that is common to SVs throughout vertebrate nervous systems, and there are three isoforms (SV2A, SV2B, and SV2C) that have differing expression patterns through development. SV2 has 12 transmembrane domains that traverse the SV membrane, seven cytoplasmic domains of varying lengths with phosphorylation sites, and six luminal domains of varying lengths with at least three glycosylation sites (Bajjalieh et al., 1994; Bartholome et al., 2017). SV2 is present at frog NMJs (Dunaevsky and Connor, 2000). At mouse NMJs, SV2A is down-regulated in motor nerve terminals on fast-twitch muscle fibers after birth whereas SV2B and SV2C are retained at nearly all NMJs into adulthood (Chakkalakal et al., 2010). SV2 binds active zone proteins including Synaptotagmin, Synaptophysin, Synaptobrevin, and Rab3A (Bennett et al., 1992a), and it was also proposed that there are ~2 copies of SV2 per SV (Takamori et al., 2006). The structure of SV2 isolated from its native tissue has not been determined but based on its primary and secondary structures, the large luminal domains are able to traverse the SV lumen several times. Thus, it is likely that the luminal portions of SV2 form the bulk of the luminal assembly of macromolecules detected in SVs at frog NMJs processed by the high-pressure freezing and freeze-substitution methods of fixation and staining (Figure 1; Harlow et al., 2013). The large cytoplasmic domains are likely components of AZM macromolecules that connect to SV membranes, such as ribs, pins, spars, booms, and top-masts, and/or non-AZM macromolecules that link SVs to other undocked SVs (Figure 1; Szule et al., 2012; Harlow et al., 2013). The nubs linking the luminal assembly to the luminal surface of the SV membrane are also likely composed of a combination of SV2 at the transitions to transmembrane regions and also the luminal portions of other SV membrane proteins, such as synaptotagmin, synaptophysin, synaptobrevin, and Rab3A, that link to SV2.

## Summary

The current report, although not exhaustive, provides a hypothesis that incorporates cellular and morphological features of synaptic active zones with biochemical mechanisms of the transient SV trafficking events that lead to neurotransmitter secretion. These events include recruiting and tethering undocked SVs to the active zone, SV docking as a directed approach to the PM, SV priming after it has docked, Ca^2+^-triggering initiated by an electrical impulse, and fusion between the SV membrane and PM to secrete neurotransmitters. Due to the quantitative characterization of AZM at frog NMJs, both at rest and during impulse-evoked synaptic activity, it is an appropriate model system for which to propose a hypothesis relating to structure, biochemistry, and function. AZM is composed of several morphologically distinct macromolecules that each play a role in the transient stages of membrane trafficking and active zone assembly/organization. In summary (see Table 2), the cation channels are proposed to be included in pegs and ribs, the SNARE proteins and SNARE auxiliary proteins are proposed to be included in ribs and pins, Rab3A and Rabphillin-3A are proposed to be included in spars and/or booms and/or top-masts, the scaffolding proteins are proposed to be included in steps and masts, and SV2 is proposed to compose the bulk of SV luminal filaments. It would be of great interest to test this model so that the function of AZM at presynaptic terminals can be understood at the molecular level.

## Data Availability Statement

The original contributions presented in the study are included in the article, further inquiries can be directed to the corresponding author.

## Author Contributions

The author confirms being the sole contributor of this work and has approved it for publication.

## Conflict of Interest

The author declares that the research was conducted in the absence of any commercial or financial relationships that could be construed as a potential conflict of interest.

## Publisher’s Note

All claims expressed in this article are solely those of the authors and do not necessarily represent those of their affiliated organizations, or those of the publisher, the editors and the reviewers. Any product that may be evaluated in this article, or claim that may be made by its manufacturer, is not guaranteed or endorsed by the publisher.

## Footnotes

^1^ http://www.wwpdb.org

## Abbreviations

NMJ: Neuromuscular junction
AZM: Active zone material
PM: Presynaptic membrane
SV: Synaptic vesicle.
